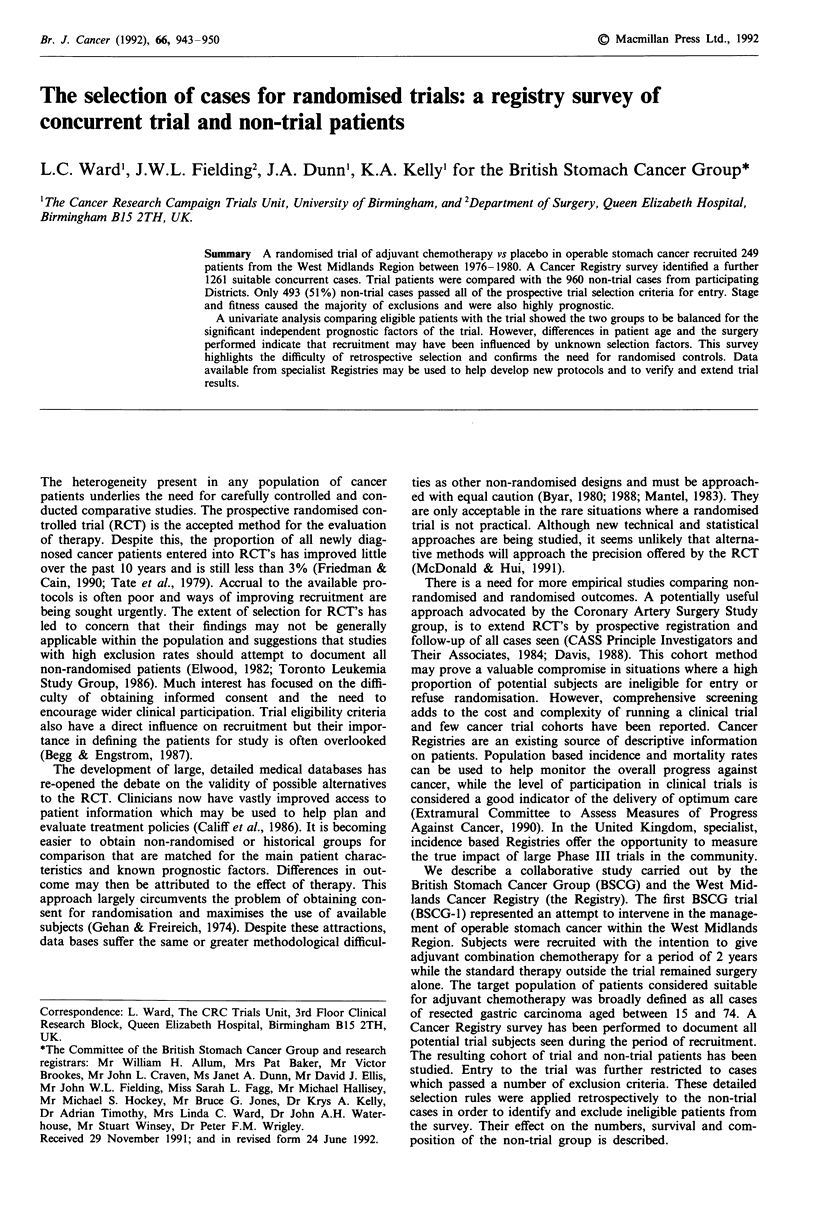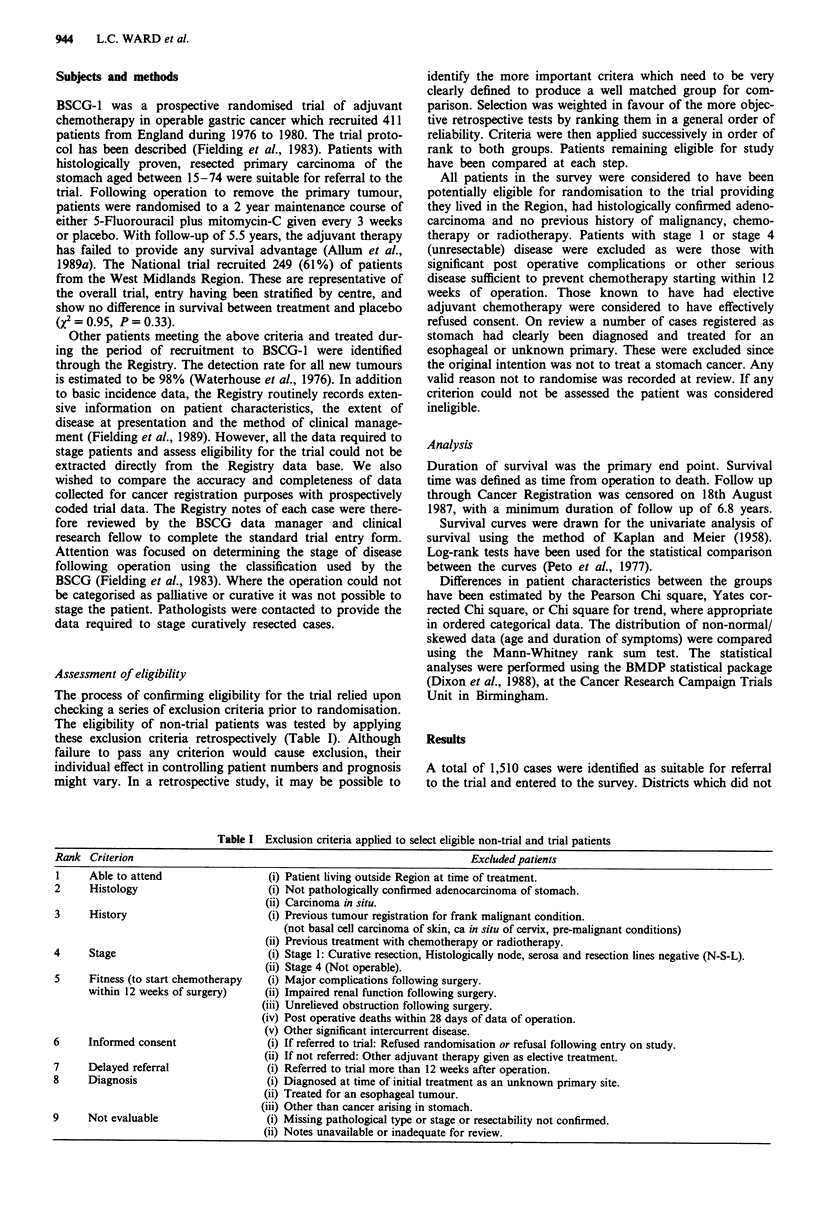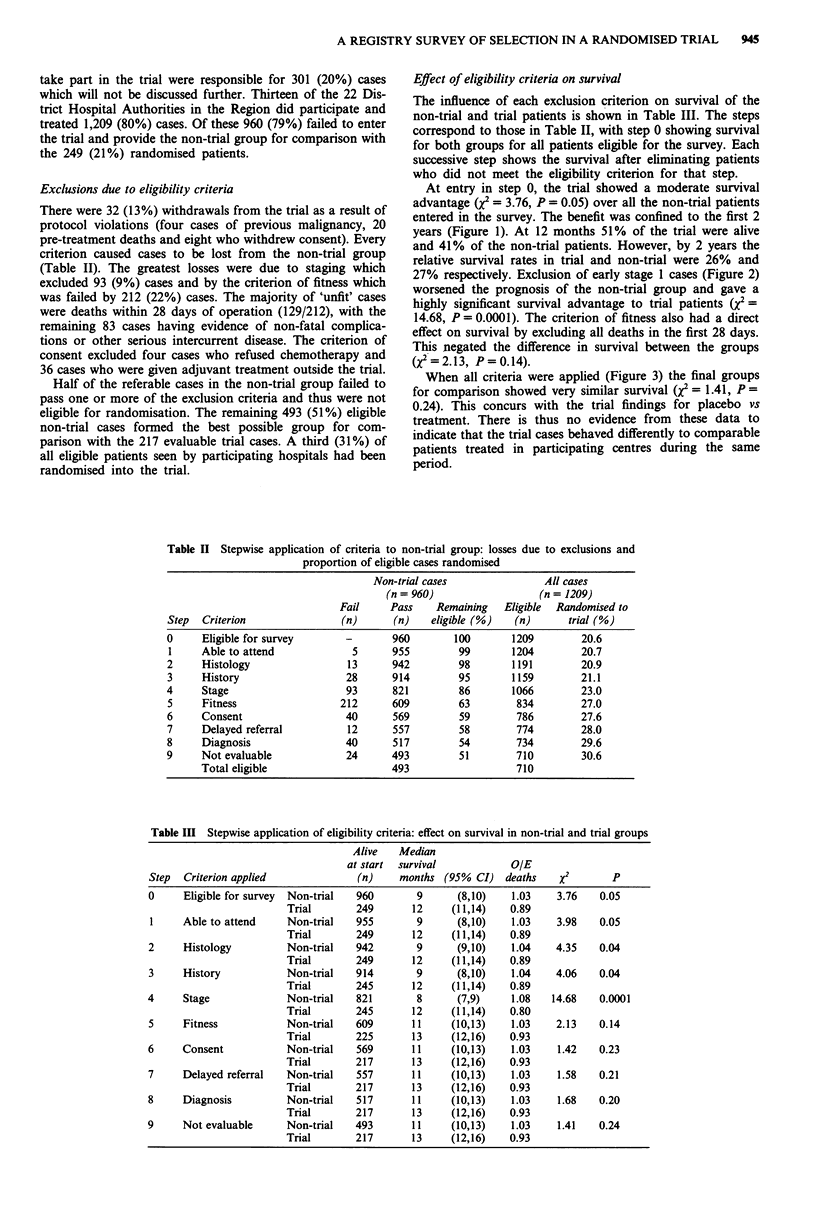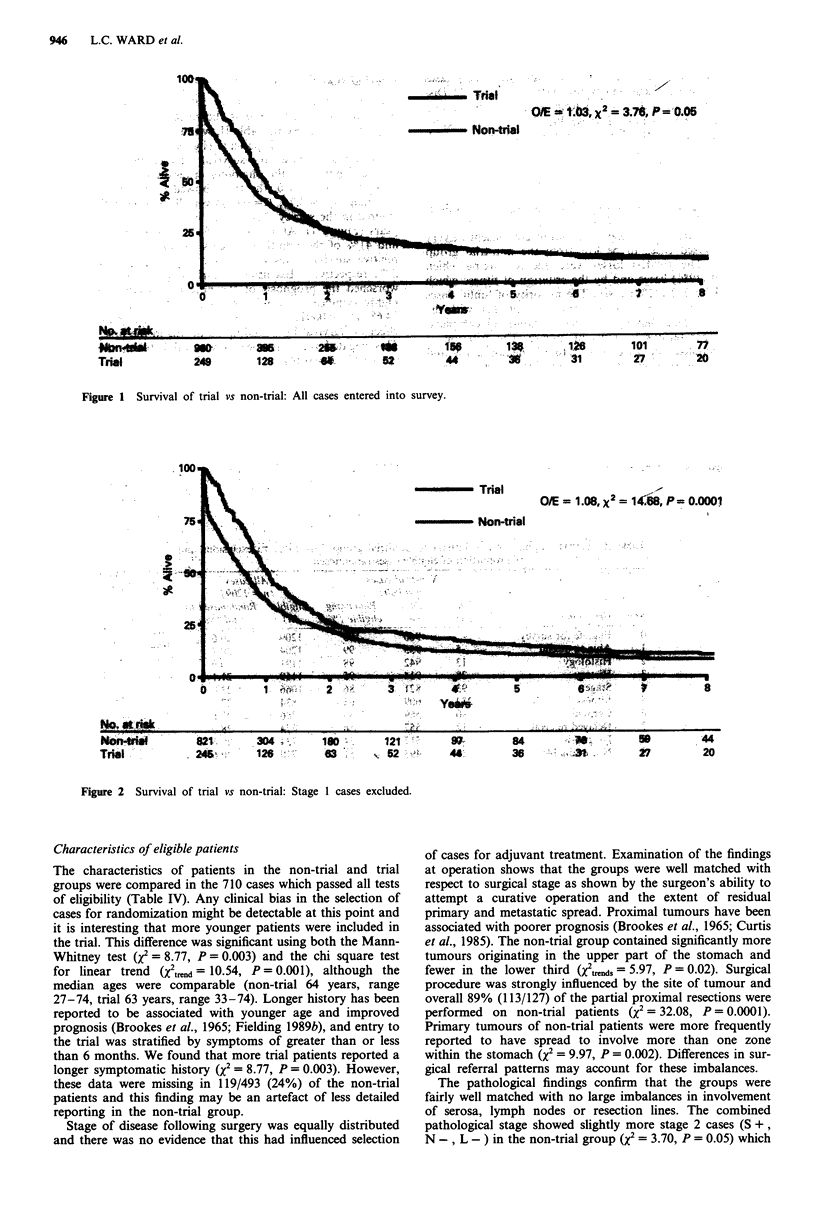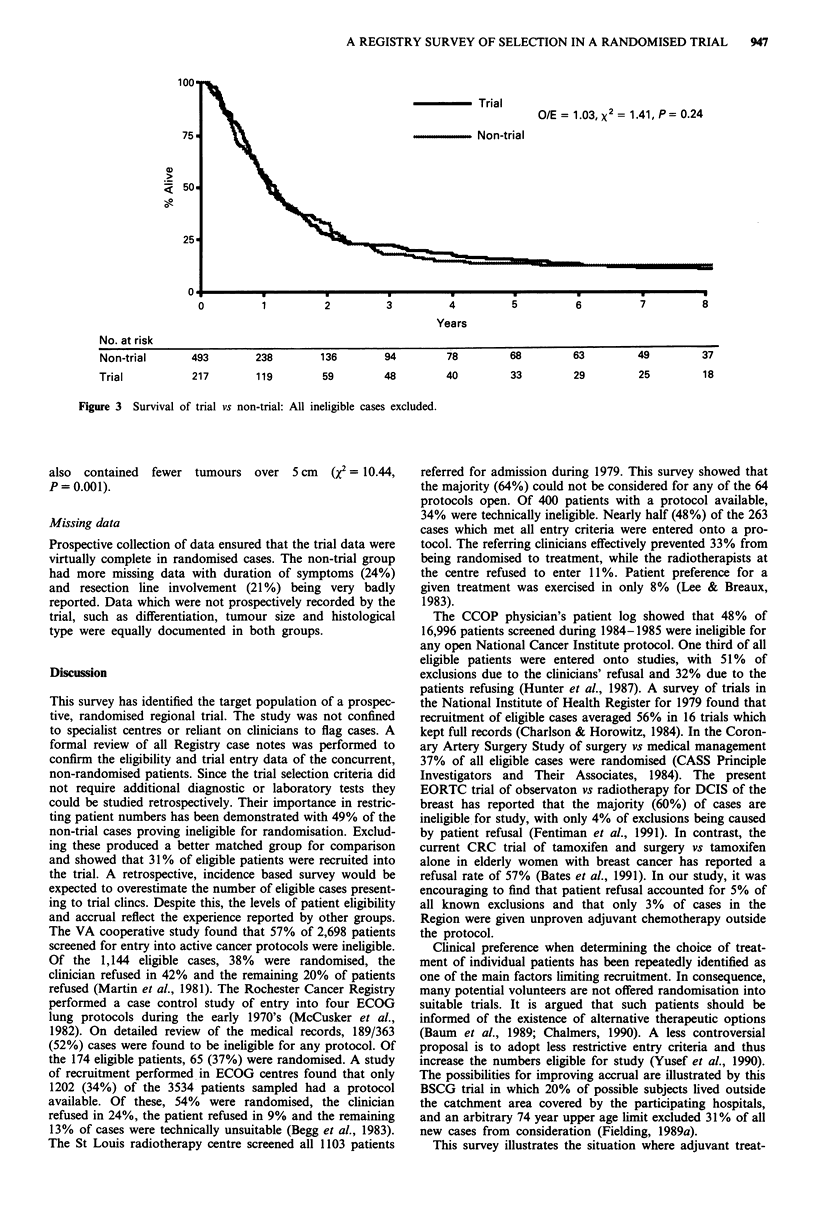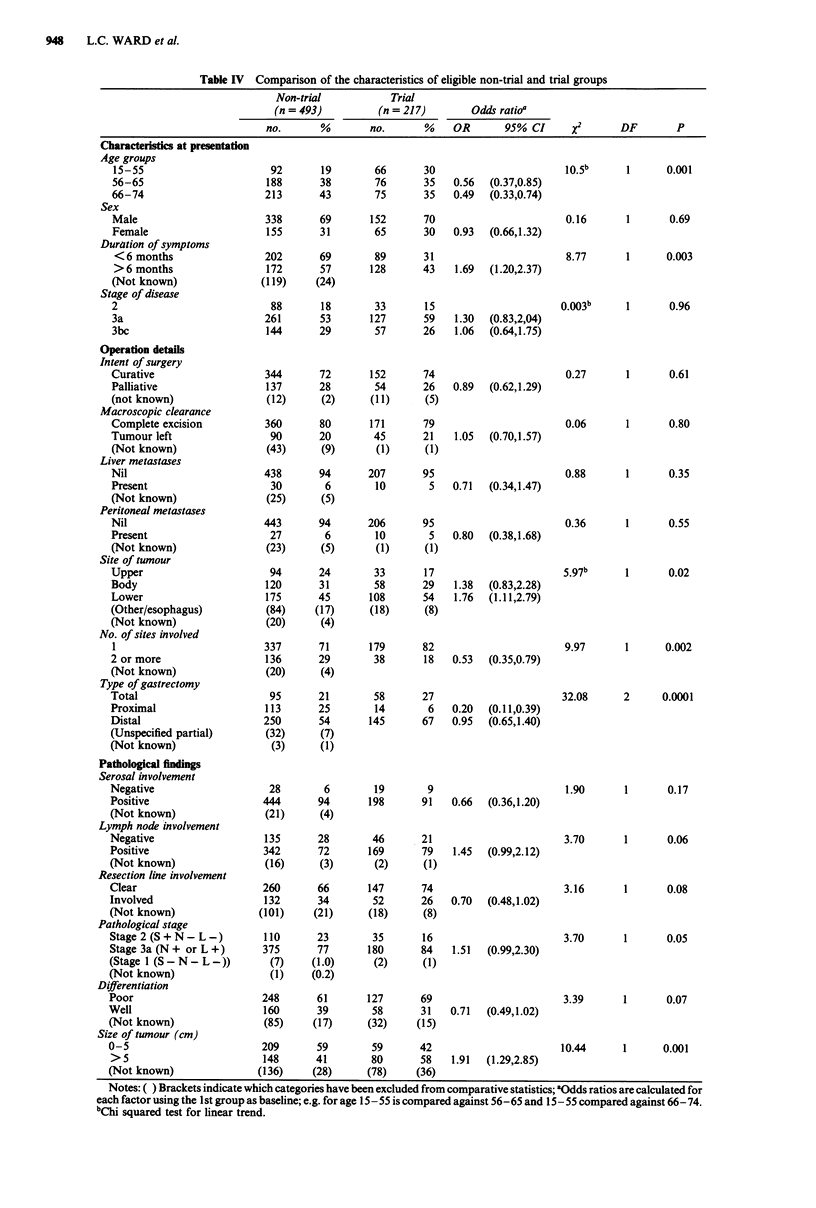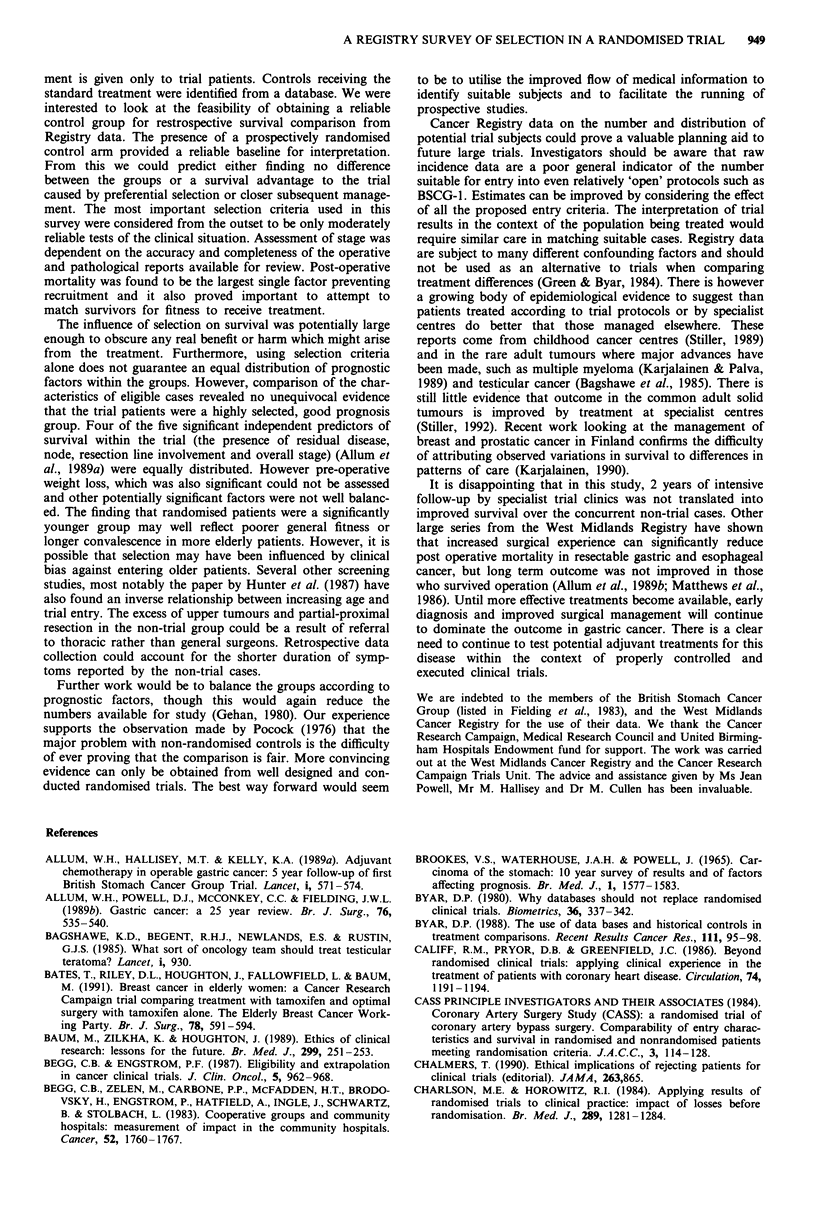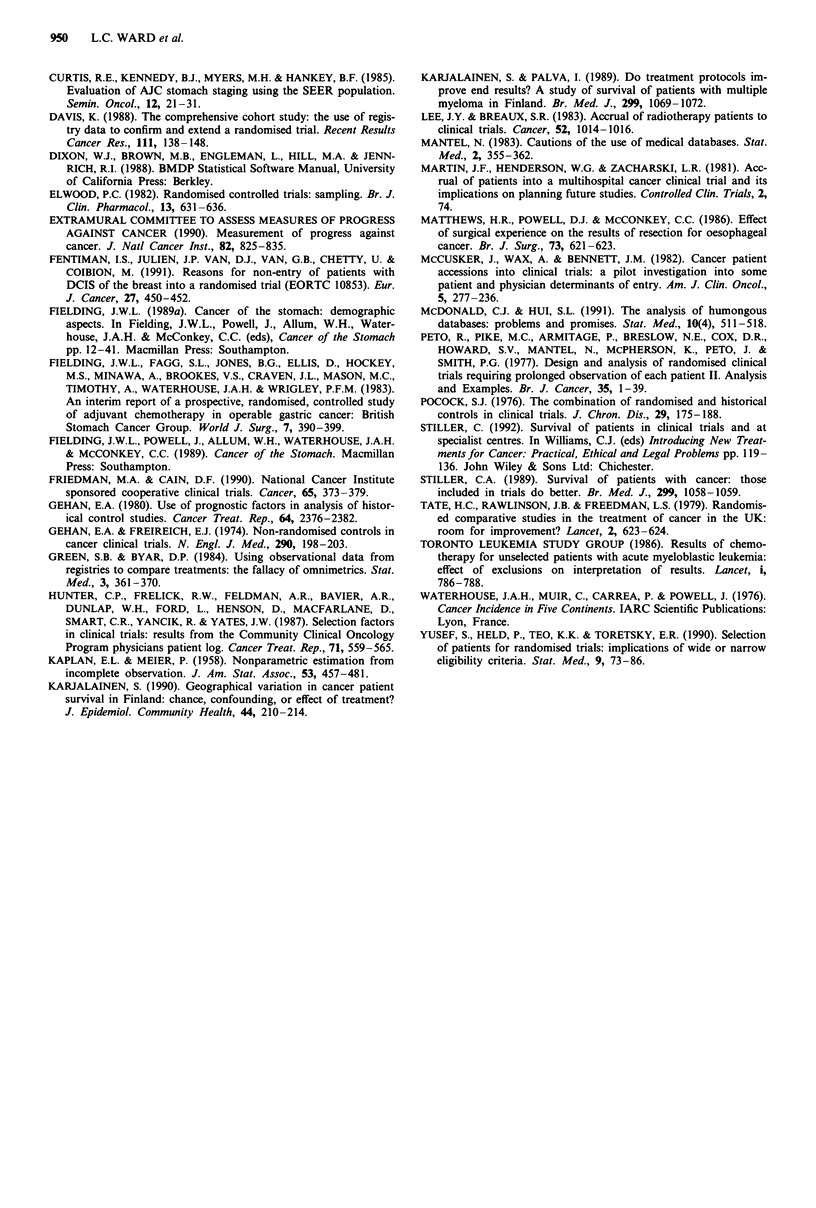# The selection of cases for randomised trials: a registry survey of concurrent trial and non-trial patients. The British Stomach Cancer Group.

**DOI:** 10.1038/bjc.1992.390

**Published:** 1992-11

**Authors:** L. C. Ward, J. W. Fielding, J. A. Dunn, K. A. Kelly

**Affiliations:** Cancer Research Campaign Trials Unit, University of Birmingham, UK.

## Abstract

A randomised trial of adjuvant chemotherapy vs placebo in operable stomach cancer recruited 249 patients from the West Midlands Region between 1976-1980. A Cancer Registry survey identified a further 1261 suitable concurrent cases. Trial patients were compared with the 960 non-trial cases from participating Districts. Only 493 (51%) non-trial cases passed all of the prospective trial selection criteria for entry. Stage and fitness caused the majority of exclusions and were also highly prognostic. A univariate analysis comparing eligible patients with the trial showed the two groups to be balanced for the significant independent prognostic factors of the trial. However, differences in patient age and the surgery performed indicate that recruitment may have been influenced by unknown selection factors. This survey highlights the difficulty of retrospective selection and confirms the need for randomised controls. Data available from specialist Registries may be used to help develop new protocols and to verify and extend trial results.


Br. J. Cancer (1992), 66, 943–950 © Macmillan Press Ltd., 1992

### The selection of cases for randomised trials: a registry survey of concurrent trial and non-trial patients

L.C. Ward[1], J.W.L. Fielding[2], J.A. Dunn[1], K.A. Kelly[1] for the British Stomach Cancer Group*

[1]The Cancer Research Campaign Trials Unit, University of Birmingham, and [2]Department of Surgery, Queen Elizabeth Hospital, Birmingham B15 2TH, UK.

**Summary** A randomised trial of adjuvant chemotherapy *vs* placebo in operable stomach cancer recruited 249 patients from the West Midlands Region between 1976–1980. A Cancer Registry survey identified a further 1261 suitable concurrent cases. Trial patients were compared with the 960 non-trial cases from participating Districts. Only 493 (51%) non-trial cases passed all of the prospective trial selection criteria for entry. Stage and fitness caused the majority of exclusions and were also highly prognostic.

A univariate analysis comparing eligible patients with the trial showed the two groups to be balanced for the significant independent prognostic factors of the trial. However, differences in patient age and the surgery performed indicate that recruitment may have been influenced by unknown selection factors. This survey highlights the difficulty of retrospective selection and confirms the need for randomised controls. Data available from specialist Registries may be used to help develop new protocols and to verify and extend trial results.

The heterogeneity present in any population of cancer patients underlies the need for carefully controlled and conducted comparative studies. The prospective randomised controlled trial (RCT) is the accepted method for the evaluation of therapy. Despite this, the proportion of all newly diagnosed cancer patients entered into RCT's has improved little over the past 10 years and is still less than 3% (Friedman & Cain, 1990; Tate *et al.*, 1979). Accrual to the available protocols is often poor and ways of improving recruitment are being sought urgently. The extent of selection for RCT's has led to concern that their findings may not be generally applicable within the population and suggestions that studies with high exclusion rates should attempt to document all non-randomised patients (Elwood, 1982; Toronto Leukemia Study Group, 1986). Much interest has focused on the difficulty of obtaining informed consent and the need to encourage wider clinical participation. Trial eligibility criteria also have a direct influence on recruitment but their importance in defining the patients for study is often overlooked (Begg & Engstrom, 1987).

The development of large, detailed medical databases has re-opened the debate on the validity of possible alternatives to the RCT. Clinicians now have vastly improved access to patient information which may be used to help plan and evaluate treatment policies (Califf *et al.*, 1986). It is becoming easier to obtain non-randomised or historical groups for comparison that are matched for the main patient characteristics and known prognostic factors. Differences in outcome may then be attributed to the effect of therapy. This approach largely circumvents the problem of obtaining consent for randomisation and maximises the use of available subjects (Gehan & Freireich, 1974). Despite these attractions, data bases suffer the same or greater methodological difficulties as other non-randomised designs and must be approached with equal caution (Byar, 1980; 1988; Mantel, 1983). They are only acceptable in the rare situations where a randomised trial is not practical. Although new technical and statistical approaches are being studied, it seems unlikely that alternative methods will approach the precision offered by the RCT (McDonald & Hui, 1991).

There is a need for more empirical studies comparing non-randomised and randomised outcomes. A potentially useful approach advocated by the Coronary Artery Surgery Study group, is to extend RCT's by prospective registration and follow-up of all cases seen (CASS Principle Investigators and Their Associates, 1984; Davis, 1988). This cohort method may prove a valuable compromise in situations where a high proportion of potential subjects are ineligible for entry or refuse randomisation. However, comprehensive screening adds to the cost and complexity of running a clinical trial and few cancer trial cohorts have been reported. Cancer Registries are an existing source of descriptive information on patients. Population based incidence and mortality rates can be used to help monitor the overall progress against cancer, while the level of participation in clinical trials is considered a good indicator of the delivery of optimum care (Extramural Committee to Assess Measures of Progress Against Cancer, 1990). In the United Kingdom, specialist, incidence based Registries offer the opportunity to measure the true impact of large Phase III trials in the community.

We describe a collaborative study carried out by the British Stomach Cancer Group (BSCG) and the West Midlands Cancer Registry (the Registry). The first BSCG trial (BSCG-1) represented an attempt to intervene in the management of operable stomach cancer within the West Midlands Region. Subjects were recruited with the intention to give adjuvant combination chemotherapy for a period of 2 years while the standard therapy outside the trial remained surgery alone. The target population of patients considered suitable for adjuvant chemotherapy was broadly defined as all cases of resected gastric carcinoma aged between 15 and 74. A Cancer Registry survey has been performed to document all potential trial subjects seen during the period of recruitment. The resulting cohort of trial and non-trial patients has been studied. Entry to the trial was further restricted to cases which passed a number of exclusion criteria. These detailed selection rules were applied retrospectively to the non-trial cases in order to identify and exclude ineligible patients from the survey. Their effect on the numbers, survival and composition of the non-trial group is described.

Correspondence: L. Ward, The CRC Trials Unit, 3rd Floor Clinical Research Block, Queen Elizabeth Hospital, Birmingham B15 2TH, UK.

*The Committee of the British Stomach Cancer Group and research registrars: Mr William H. Allum, Mrs Pat Baker, Mr Victor Brookes, Mr John L. Craven, Ms Janet A. Dunn, Mr David J. Ellis, Mr John W.L. Fielding, Miss Sarah L. Fagg, Mr Michael Hallisey, Mr Michael S. Hockey, Mr Bruce G. Jones, Dr Krys A. Kelly, Dr Adrian Timothy, Mrs Linda C. Ward, Dr John A.H. Waterhouse, Mr Stuart Winsey, Dr Peter F.M. Wrigley.

Received 29 November 1991; and in revised form 24 June 1992.

### Subjects and methods

BSCG-1 was a prospective randomised trial of adjuvant chemotherapy in operable gastric cancer which recruited 411 patients from England during 1976 to 1980. The trial protocol has been described (Fielding *et al.*, 1983). Patients with histologically proven, resected primary carcinoma of the stomach aged between 15–74 were suitable for referral to the trial. Following operation to remove the primary tumour, patients were randomised to a 2 year maintenance course of either 5-Fluorouracil plus mitomycin-C given every 3 weeks or placebo. With follow-up of 5.5 years, the adjuvant therapy has failed to provide any survival advantage (Allum *et al.*, 1989*a*). The National trial recruited 249 (61%) of patients from the West Midlands Region. These are representative of the overall trial, entry having been stratified by centre, and show no difference in survival between treatment and placebo ($\chi^2 = 0.95$, $P = 0.33$).

Other patients meeting the above criteria and treated during the period of recruitment to BSCG-1 were identified through the Registry. The detection rate for all new tumours is estimated to be 98% (Waterhouse *et al.*, 1976). In addition to basic incidence data, the Registry routinely records extensive information on patient characteristics, the extent of disease at presentation and the method of clinical management (Fielding *et al.*, 1989). However, all the data required to stage patients and assess eligibility for the trial could not be extracted directly from the Registry data base. We also wished to compare the accuracy and completeness of data collected for cancer registration purposes with prospectively coded trial data. The Registry notes of each case were therefore reviewed by the BSCG data manager and clinical research fellow to complete the standard trial entry form. Attention was focused on determining the stage of disease following operation using the classification used by the BSCG (Fielding *et al.*, 1983). Where the operation could not be categorised as palliative or curative it was not possible to stage the patient. Pathologists were contacted to provide the data required to stage curatively resected cases.

### *Assessment of eligibility*

The process of confirming eligibility for the trial relied upon checking a series of exclusion criteria prior to randomisation. The eligibility of non-trial patients was tested by applying these exclusion criteria retrospectively (Table I). Although failure to pass any criterion would cause exclusion, their individual effect in controlling patient numbers and prognosis might vary. In a retrospective study, it may be possible to identify the more important critera which need to be very clearly defined to produce a well matched group for comparison. Selection was weighted in favour of the more objective retrospective tests by ranking them in a general order of reliability. Criteria were then applied successively in order of rank to both groups. Patients remaining eligible for study have been compared at each step.

All patients in the survey were considered to have been potentially eligible for randomisation to the trial providing they lived in the Region, had histologically confirmed adenocarcinoma and no previous history of malignancy, chemotherapy or radiotherapy. Patients with stage 1 or stage 4 (unresectable) disease were excluded as were those with significant post operative complications or other serious disease sufficient to prevent chemotherapy starting within 12 weeks of operation. Those known to have had elective adjuvant chemotherapy were considered to have effectively refused consent. On review a number of cases registered as stomach had clearly been diagnosed and treated for an esophageal or unknown primary. These were excluded since the original intention was not to treat a stomach cancer. Any valid reason not to randomise was recorded at review. If any criterion could not be assessed the patient was considered ineligible.

### *Analysis*

Duration of survival was the primary end point. Survival time was defined as time from operation to death. Follow up through Cancer Registration was censored on 18th August 1987, with a minimum duration of follow up of 6.8 years.

Survival curves were drawn for the univariate analysis of survival using the method of Kaplan and Meier (1958). Log-rank tests have been used for the statistical comparison between the curves (Peto *et al.*, 1977).

Differences in patient characteristics between the groups have been estimated by the Pearson Chi square, Yates corrected Chi square, or Chi square for trend, where appropriate in ordered categorical data. The distribution of non-normal/skewed data (age and duration of symptoms) were compared using the Mann-Whitney rank sum test. The statistical analyses were performed using the BMDP statistical package (Dixon *et al.*, 1988), at the Cancer Research Campaign Trials Unit in Birmingham.

### Results

A total of 1,510 cases were identified as suitable for referral to the trial and entered to the survey. Districts which did not

**Table I** Exclusion criteria applied to select eligible non-trial and trial patients

| *Rank* | *Criterion* | *Excluded patients* |
|---|---|---|
| 1 | Able to attend | (i) Patient living outside Region at time of treatment. |
| 2 | Histology | (i) Not pathologically confirmed adenocarcinoma of stomach. |
| | | (ii) Carcinoma *in situ*. |
| 3 | History | (i) Previous tumour registration for frank malignant condition. (not basal cell carcinoma of skin, ca *in situ* of cervix, pre-malignant conditions) |
| | | (ii) Previous treatment with chemotherapy or radiotherapy. |
| 4 | Stage | (i) Stage 1: Curative resection, Histologically node, serosa and resection lines negative (N-S-L). |
| | | (ii) Stage 4 (Not operable). |
| 5 | Fitness (to start chemotherapy within 12 weeks of surgery) | (i) Major complications following surgery. |
| | | (ii) Impaired renal function following surgery. |
| | | (iii) Unrelieved obstruction following surgery. |
| | | (iv) Post operative deaths within 28 days of data of operation. |
| | | (v) Other significant intercurrent disease. |
| 6 | Informed consent | (i) If referred to trial: Refused randomisation *or* refusal following entry on study. |
| | | (ii) If not referred: Other adjuvant therapy given as elective treatment. |
| 7 | Delayed referral | (i) Referred to trial more than 12 weeks after operation. |
| 8 | Diagnosis | (i) Diagnosed at time of initial treatment as an unknown primary site. |
| | | (ii) Treated for an esophageal tumour. |
| | | (iii) Other than cancer arising in stomach. |
| 9 | Not evaluable | (i) Missing pathological type or stage or resectability not confirmed. |
| | | (ii) Notes unavailable or inadequate for review. |

take part in the trial were responsible for 301 (20%) cases which will not be discussed further. Thirteen of the 22 District Hospital Authorities in the Region did participate and treated 1,209 (80%) cases. Of these 960 (79%) failed to enter the trial and provide the non-trial group for comparison with the 249 (21%) randomised patients.

*Exclusions due to eligibility criteria*

There were 32 (13%) withdrawals from the trial as a result of protocol violations (four cases of previous malignancy, 20 pre-treatment deaths and eight who withdrew consent). Every criterion caused cases to be lost from the non-trial group (Table II). The greatest losses were due to staging which excluded 93 (9%) cases and by the criterion of fitness which was failed by 212 (22%) cases. The majority of 'unfit' cases were deaths within 28 days of operation (129/212), with the remaining 83 cases having evidence of non-fatal complications or other serious intercurrent disease. The criterion of consent excluded four cases who refused chemotherapy and 36 cases who were given adjuvant treatment outside the trial.

Half of the referable cases in the non-trial group failed to pass one or more of the exclusion criteria and thus were not eligible for randomisation. The remaining 493 (51%) eligible non-trial cases formed the best possible group for comparison with the 217 evaluable trial cases. A third (31%) of all eligible patients seen by participating hospitals had been randomised into the trial.

*Effect of eligibility criteria on survival*

The influence of each exclusion criterion on survival of the non-trial and trial patients is shown in Table III. The steps correspond to those in Table II, with step 0 showing survival for both groups for all patients eligible for the survey. Each successive step shows the survival after eliminating patients who did not meet the eligibility criterion for that step.

At entry in step 0, the trial showed a moderate survival advantage ($\chi^2 = 3.76$, $P = 0.05$) over all the non-trial patients entered in the survey. The benefit was confined to the first 2 years (Figure 1). At 12 months 51% of the trial were alive and 41% of the non-trial patients. However, by 2 years the relative survival rates in trial and non-trial were 26% and 27% respectively. Exclusion of early stage 1 cases (Figure 2) worsened the prognosis of the non-trial group and gave a highly significant survival advantage to trial patients ($\chi^2 = 14.68$, $P = 0.0001$). The criterion of fitness also had a direct effect on survival by excluding all deaths in the first 28 days. This negated the difference in survival between the groups ($\chi^2 = 2.13$, $P = 0.14$).

When all criteria were applied (Figure 3) the final groups for comparison showed very similar survival ($\chi^2 = 1.41$, $P = 0.24$). This concurs with the trial findings for placebo *vs* treatment. There is thus no evidence from these data to indicate that the trial cases behaved differently to comparable patients treated in participating centres during the same period.

**Table II** Stepwise application of criteria to non-trial group: losses due to exclusions and proportion of eligible cases randomised

| | | Non-trial cases (n = 960) | | | All cases (n = 1209) | |
|---|---|---|---|---|---|---|
| *Step* | *Criterion* | *Fail (n)* | *Pass (n)* | *Remaining eligible (%)* | *Eligible (n)* | *Randomised to trial (%)* |
| 0 | Eligible for survey | – | 960 | 100 | 1209 | 20.6 |
| 1 | Able to attend | 5 | 955 | 99 | 1204 | 20.7 |
| 2 | Histology | 13 | 942 | 98 | 1191 | 20.9 |
| 3 | History | 28 | 914 | 95 | 1159 | 21.1 |
| 4 | Stage | 93 | 821 | 86 | 1066 | 23.0 |
| 5 | Fitness | 212 | 609 | 63 | 834 | 27.0 |
| 6 | Consent | 40 | 569 | 59 | 786 | 27.6 |
| 7 | Delayed referral | 12 | 557 | 58 | 774 | 28.0 |
| 8 | Diagnosis | 40 | 517 | 54 | 734 | 29.6 |
| 9 | Not evaluable | 24 | 493 | 51 | 710 | 30.6 |
| | Total eligible | | 493 | | 710 | |

**Table III** Stepwise application of eligibility criteria: effect on survival in non-trial and trial groups

| *Step* | *Criterion applied* | | *Alive at start (n)* | *Median survival months* | *(95% CI)* | *O/E deaths* | $\chi^2$ | *P* |
|---|---|---|---|---|---|---|---|---|
| 0 | Eligible for survey | Non-trial | 960 | 9 | (8,10) | 1.03 | 3.76 | 0.05 |
| | | Trial | 249 | 12 | (11,14) | 0.89 | | |
| 1 | Able to attend | Non-trial | 955 | 9 | (8,10) | 1.03 | 3.98 | 0.05 |
| | | Trial | 249 | 12 | (11,14) | 0.89 | | |
| 2 | Histology | Non-trial | 942 | 9 | (9,10) | 1.04 | 4.35 | 0.04 |
| | | Trial | 249 | 12 | (11,14) | 0.89 | | |
| 3 | History | Non-trial | 914 | 9 | (8,10) | 1.04 | 4.06 | 0.04 |
| | | Trial | 245 | 12 | (11,14) | 0.89 | | |
| 4 | Stage | Non-trial | 821 | 8 | (7,9) | 1.08 | 14.68 | 0.0001 |
| | | Trial | 245 | 12 | (11,14) | 0.80 | | |
| 5 | Fitness | Non-trial | 609 | 11 | (10,13) | 1.03 | 2.13 | 0.14 |
| | | Trial | 225 | 13 | (12,16) | 0.93 | | |
| 6 | Consent | Non-trial | 569 | 11 | (10,13) | 1.03 | 1.42 | 0.23 |
| | | Trial | 217 | 13 | (12,16) | 0.93 | | |
| 7 | Delayed referral | Non-trial | 557 | 11 | (10,13) | 1.03 | 1.58 | 0.21 |
| | | Trial | 217 | 13 | (12,16) | 0.93 | | |
| 8 | Diagnosis | Non-trial | 517 | 11 | (10,13) | 1.03 | 1.68 | 0.20 |
| | | Trial | 217 | 13 | (12,16) | 0.93 | | |
| 9 | Not evaluable | Non-trial | 493 | 11 | (10,13) | 1.03 | 1.41 | 0.24 |
| | | Trial | 217 | 13 | (12,16) | 0.93 | | |

| No. at risk | | | | | | | | | |
|---|---|---|---|---|---|---|---|---|---|
| Non-trial | 960 | 395 | 255 | 188 | 156 | 138 | 126 | 101 | 77 |
| Trial | 249 | 128 | 64 | 52 | 44 | 36 | 31 | 27 | 20 |

**Figure 1** Survival of trial *vs* non-trial: All cases entered into survey.

| No. at risk | | | | | | | | | |
|---|---|---|---|---|---|---|---|---|---|
| Non-trial | 821 | 304 | 180 | 121 | 97 | 84 | 76 | 59 | 44 |
| Trial | 245 | 126 | 63 | 52 | 44 | 36 | 31 | 27 | 20 |

**Figure 2** Survival of trial *vs* non-trial: Stage 1 cases excluded.

*Characteristics of eligible patients*

The characteristics of patients in the non-trial and trial groups were compared in the 710 cases which passed all tests of eligibility (Table IV). Any clinical bias in the selection of cases for randomization might be detectable at this point and it is interesting that more younger patients were included in the trial. This difference was significant using both the Mann-Whitney test ($\chi^2 = 8.77$, $P = 0.003$) and the chi square test for linear trend ($\chi^2_{trend} = 10.54$, $P = 0.001$), although the median ages were comparable (non-trial 64 years, range 27–74, trial 63 years, range 33–74). Longer history has been reported to be associated with younger age and improved prognosis (Brookes *et al.*, 1965; Fielding 1989*b*), and entry to the trial was stratified by symptoms of greater than or less than 6 months. We found that more trial patients reported a longer symptomatic history ($\chi^2 = 8.77$, $P = 0.003$). However, these data were missing in 119/493 (24%) of the non-trial patients and this finding may be an artefact of less detailed reporting in the non-trial group.

Stage of disease following surgery was equally distributed and there was no evidence that this had influenced selection of cases for adjuvant treatment. Examination of the findings at operation shows that the groups were well matched with respect to surgical stage as shown by the surgeon's ability to attempt a curative operation and the extent of residual primary and metastatic spread. Proximal tumours have been associated with poorer prognosis (Brookes *et al.*, 1965; Curtis *et al.*, 1985). The non-trial group contained significantly more tumours originating in the upper part of the stomach and fewer in the lower third ($\chi^2_{trends} = 5.97$, $P = 0.02$). Surgical procedure was strongly influenced by the site of tumour and overall 89% (113/127) of the partial proximal resections were performed on non-trial patients ($\chi^2 = 32.08$, $P = 0.0001$). Primary tumours of non-trial patients were more frequently reported to have spread to involve more than one zone within the stomach ($\chi^2 = 9.97$, $P = 0.002$). Differences in surgical referral patterns may account for these imbalances.

The pathological findings confirm that the groups were fairly well matched with no large imbalances in involvement of serosa, lymph nodes or resection lines. The combined pathological stage showed slightly more stage 2 cases (S +, N –, L –) in the non-trial group ($\chi^2 = 3.70$, $P = 0.05$) which

| No. at risk | | | | | | | | | |
|---|---|---|---|---|---|---|---|---|---|
| Non-trial | 493 | 238 | 136 | 94 | 78 | 68 | 63 | 49 | 37 |
| Trial | 217 | 119 | 59 | 48 | 40 | 33 | 29 | 25 | 18 |

**Figure 3** Survival of trial *vs* non-trial: All ineligible cases excluded.

also contained fewer tumours over 5 cm ($\chi^2 = 10.44$, $P = 0.001$).

### *Missing data*

Prospective collection of data ensured that the trial data were virtually complete in randomised cases. The non-trial group had more missing data with duration of symptoms (24%) and resection line involvement (21%) being very badly reported. Data which were not prospectively recorded by the trial, such as differentiation, tumour size and histological type were equally documented in both groups.

### Discussion

This survey has identified the target population of a prospective, randomised regional trial. The study was not confined to specialist centres or reliant on clinicians to flag cases. A formal review of all Registry case notes was performed to confirm the eligibility and trial entry data of the concurrent, non-randomised patients. Since the trial selection criteria did not require additional diagnostic or laboratory tests they could be studied retrospectively. Their importance in restricting patient numbers has been demonstrated with 49% of the non-trial cases proving ineligible for randomisation. Excluding these produced a better matched group for comparison and showed that 31% of eligible patients were recruited into the trial. A retrospective, incidence based survey would be expected to overestimate the number of eligible cases presenting to trial clincs. Despite this, the levels of patient eligibility and accrual reflect the experience reported by other groups. The VA cooperative study found that 57% of 2,698 patients screened for entry into active cancer protocols were ineligible. Of the 1,144 eligible cases, 38% were randomised, the clinician refused in 42% and the remaining 20% of patients refused (Martin *et al.*, 1981). The Rochester Cancer Registry performed a case control study of entry into four ECOG lung protocols during the early 1970's (McCusker *et al.*, 1982). On detailed review of the medical records, 189/363 (52%) cases were found to be ineligible for any protocol. Of the 174 eligible patients, 65 (37%) were randomised. A study of recruitment performed in ECOG centres found that only 1202 (34%) of the 3534 patients sampled had a protocol available. Of these, 54% were randomised, the clinician refused in 24%, the patient refused in 9% and the remaining 13% of cases were technically unsuitable (Begg *et al.*, 1983). The St Louis radiotherapy centre screened all 1103 patients referred for admission during 1979. This survey showed that the majority (64%) could not be considered for any of the 64 protocols open. Of 400 patients with a protocol available, 34% were technically ineligible. Nearly half (48%) of the 263 cases which met all entry criteria were entered onto a protocol. The referring clinicians effectively prevented 33% from being randomised to treatment, while the radiotherapists at the centre refused to enter 11%. Patient preference for a given treatment was exercised in only 8% (Lee & Breaux, 1983).

The CCOP physician's patient log showed that 48% of 16,996 patients screened during 1984–1985 were ineligible for any open National Cancer Institute protocol. One third of all eligible patients were entered onto studies, with 51% of exclusions due to the clinicians' refusal and 32% due to the patients refusing (Hunter *et al.*, 1987). A survey of trials in the National Institute of Health Register for 1979 found that recruitment of eligible cases averaged 56% in 16 trials which kept full records (Charlson & Horowitz, 1984). In the Coronary Artery Surgery Study of surgery *vs* medical management 37% of all eligible cases were randomised (CASS Principle Investigators and Their Associates, 1984). The present EORTC trial of observaton *vs* radiotherapy for DCIS of the breast has reported that the majority (60%) of cases are ineligible for study, with only 4% of exclusions being caused by patient refusal (Fentiman *et al.*, 1991). In contrast, the current CRC trial of tamoxifen and surgery *vs* tamoxifen alone in elderly women with breast cancer has reported a refusal rate of 57% (Bates *et al.*, 1991). In our study, it was encouraging to find that patient refusal accounted for 5% of all known exclusions and that only 3% of cases in the Region were given unproven adjuvant chemotherapy outside the protocol.

Clinical preference when determining the choice of treatment of individual patients has been repeatedly identified as one of the main factors limiting recruitment. In consequence, many potential volunteers are not offered randomisation into suitable trials. It is argued that such patients should be informed of the existence of alternative therapeutic options (Baum *et al.*, 1989; Chalmers, 1990). A less controversial proposal is to adopt less restrictive entry criteria and thus increase the numbers eligible for study (Yusef *et al.*, 1990). The possibilities for improving accrual are illustrated by this BSCG trial in which 20% of possible subjects lived outside the catchment area covered by the participating hospitals, and an arbitrary 74 year upper age limit excluded 31% of all new cases from consideration (Fielding, 1989*a*).

This survey illustrates the situation where adjuvant treat-

**Table IV** Comparison of the characteristics of eligible non-trial and trial groups

| | *Non-trial (n = 493)* | | *Trial (n = 217)* | | *Odds ratio*[a] | | | | |
|---|---|---|---|---|---|---|---|---|---|
| | *no.* | *%* | *no.* | *%* | *OR* | *95% CI* | $\chi^2$ | *DF* | *P* |
| **Characteristics at presentation** | | | | | | | | | |
| *Age groups* | | | | | | | | | |
| 15–55 | 92 | 19 | 66 | 30 | | | 10.5[b] | 1 | 0.001 |
| 56–65 | 188 | 38 | 76 | 35 | 0.56 | (0.37,0.85) | | | |
| 66–74 | 213 | 43 | 75 | 35 | 0.49 | (0.33,0.74) | | | |
| *Sex* | | | | | | | | | |
| Male | 338 | 69 | 152 | 70 | | | 0.16 | 1 | 0.69 |
| Female | 155 | 31 | 65 | 30 | 0.93 | (0.66,1.32) | | | |
| *Duration of symptoms* | | | | | | | | | |
| <6 months | 202 | 69 | 89 | 31 | | | 8.77 | 1 | 0.003 |
| >6 months | 172 | 57 | 128 | 43 | 1.69 | (1.20,2.37) | | | |
| (Not known) | (119) | (24) | | | | | | | |
| *Stage of disease* | | | | | | | | | |
| 2 | 88 | 18 | 33 | 15 | | | 0.003[b] | 1 | 0.96 |
| 3a | 261 | 53 | 127 | 59 | 1.30 | (0.83,2,04) | | | |
| 3bc | 144 | 29 | 57 | 26 | 1.06 | (0.64,1.75) | | | |
| **Operation details** | | | | | | | | | |
| *Intent of surgery* | | | | | | | | | |
| Curative | 344 | 72 | 152 | 74 | | | 0.27 | 1 | 0.61 |
| Palliative | 137 | 28 | 54 | 26 | 0.89 | (0.62,1.29) | | | |
| (not known) | (12) | (2) | (11) | (5) | | | | | |
| *Macroscopic clearance* | | | | | | | | | |
| Complete excision | 360 | 80 | 171 | 79 | | | 0.06 | 1 | 0.80 |
| Tumour left | 90 | 20 | 45 | 21 | 1.05 | (0.70,1.57) | | | |
| (Not known) | (43) | (9) | (1) | (1) | | | | | |
| *Liver metastases* | | | | | | | | | |
| Nil | 438 | 94 | 207 | 95 | | | 0.88 | 1 | 0.35 |
| Present | 30 | 6 | 10 | 5 | 0.71 | (0.34,1.47) | | | |
| (Not known) | (25) | (5) | | | | | | | |
| *Peritoneal metastases* | | | | | | | | | |
| Nil | 443 | 94 | 206 | 95 | | | 0.36 | 1 | 0.55 |
| Present | 27 | 6 | 10 | 5 | 0.80 | (0.38,1.68) | | | |
| (Not known) | (23) | (5) | (1) | (1) | | | | | |
| *Site of tumour* | | | | | | | | | |
| Upper | 94 | 24 | 33 | 17 | | | 5.97[b] | 1 | 0.02 |
| Body | 120 | 31 | 58 | 29 | 1.38 | (0.83,2.28) | | | |
| Lower | 175 | 45 | 108 | 54 | 1.76 | (1.11,2.79) | | | |
| (Other/esophagus) | (84) | (17) | (18) | (8) | | | | | |
| (Not known) | (20) | (4) | | | | | | | |
| *No. of sites involved* | | | | | | | | | |
| 1 | 337 | 71 | 179 | 82 | | | 9.97 | 1 | 0.002 |
| 2 or more | 136 | 29 | 38 | 18 | 0.53 | (0.35,0.79) | | | |
| (Not known) | (20) | (4) | | | | | | | |
| *Type of gastrectomy* | | | | | | | | | |
| Total | 95 | 21 | 58 | 27 | | | 32.08 | 2 | 0.0001 |
| Proximal | 113 | 25 | 14 | 6 | 0.20 | (0.11,0.39) | | | |
| Distal | 250 | 54 | 145 | 67 | 0.95 | (0.65,1.40) | | | |
| (Unspecified partial) | (32) | (7) | | | | | | | |
| (Not known) | (3) | (1) | | | | | | | |
| **Pathological findings** | | | | | | | | | |
| *Serosal involvement* | | | | | | | | | |
| Negative | 28 | 6 | 19 | 9 | | | 1.90 | 1 | 0.17 |
| Positive | 444 | 94 | 198 | 91 | 0.66 | (0.36,1.20) | | | |
| (Not known) | (21) | (4) | | | | | | | |
| *Lymph node involvement* | | | | | | | | | |
| Negative | 135 | 28 | 46 | 21 | | | 3.70 | 1 | 0.06 |
| Positive | 342 | 72 | 169 | 79 | 1.45 | (0.99,2.12) | | | |
| (Not known) | (16) | (3) | (2) | (1) | | | | | |
| *Resection line involvement* | | | | | | | | | |
| Clear | 260 | 66 | 147 | 74 | | | 3.16 | 1 | 0.08 |
| Involved | 132 | 34 | 52 | 26 | 0.70 | (0.48,1.02) | | | |
| (Not known) | (101) | (21) | (18) | (8) | | | | | |
| *Pathological stage* | | | | | | | | | |
| Stage 2 (S + N − L −) | 110 | 23 | 35 | 16 | | | 3.70 | 1 | 0.05 |
| Stage 3a (N + or L +) | 375 | 77 | 180 | 84 | 1.51 | (0.99,2.30) | | | |
| (Stage 1 (S − N − L −)) | (7) | (1.0) | (2) | (1) | | | | | |
| (Not known) | (1) | (0.2) | | | | | | | |
| *Differentiation* | | | | | | | | | |
| Poor | 248 | 61 | 127 | 69 | | | 3.39 | 1 | 0.07 |
| Well | 160 | 39 | 58 | 31 | 0.71 | (0.49,1.02) | | | |
| (Not known) | (85) | (17) | (32) | (15) | | | | | |
| *Size of tumour (cm)* | | | | | | | | | |
| 0–5 | 209 | 59 | 59 | 42 | | | 10.44 | 1 | 0.001 |
| >5 | 148 | 41 | 80 | 58 | 1.91 | (1.29,2.85) | | | |
| (Not known) | (136) | (28) | (78) | (36) | | | | | |

Notes: ( ) Brackets indicate which categories have been excluded from comparative statistics; [a]Odds ratios are calculated for each factor using the 1st group as baseline; e.g. for age 15–55 is compared against 56–65 and 15–55 compared against 66–74. [b]Chi squared test for linear trend.

ment is given only to trial patients. Controls receiving the standard treatment were identified from a database. We were interested to look at the feasibility of obtaining a reliable control group for restrospective survival comparison from Registry data. The presence of a prospectively randomised control arm provided a reliable baseline for interpretation. From this we could predict either finding no difference between the groups or a survival advantage to the trial caused by preferential selection or closer subsequent management. The most important selection criteria used in this survey were considered from the outset to be only moderately reliable tests of the clinical situation. Assessment of stage was dependent on the accuracy and completeness of the operative and pathological reports available for review. Post-operative mortality was found to be the largest single factor preventing recruitment and it also proved important to attempt to match survivors for fitness to receive treatment.

The influence of selection on survival was potentially large enough to obscure any real benefit or harm which might arise from the treatment. Furthermore, using selection criteria alone does not guarantee an equal distribution of prognostic factors within the groups. However, comparison of the characteristics of eligible cases revealed no unequivocal evidence that the trial patients were a highly selected, good prognosis group. Four of the five significant independent predictors of survival within the trial (the presence of residual disease, node, resection line involvement and overall stage) (Allum *et al.*, 1989*a*) were equally distributed. However pre-operative weight loss, which was also significant could not be assessed and other potentially significant factors were not well balanced. The finding that randomised patients were a significantly younger group may well reflect poorer general fitness or longer convalescence in more elderly patients. However, it is possible that selection may have been influenced by clinical bias against entering older patients. Several other screening studies, most notably the paper by Hunter *et al.* (1987) have also found an inverse relationship between increasing age and trial entry. The excess of upper tumours and partial-proximal resection in the non-trial group could be a result of referral to thoracic rather than general surgeons. Retrospective data collection could account for the shorter duration of symptoms reported by the non-trial cases.

Further work would be to balance the groups according to prognostic factors, though this would again reduce the numbers available for study (Gehan, 1980). Our experience supports the observation made by Pocock (1976) that the major problem with non-randomised controls is the difficulty of ever proving that the comparison is fair. More convincing evidence can only be obtained from well designed and conducted randomised trials. The best way forward would seem to be to utilise the improved flow of medical information to identify suitable subjects and to facilitate the running of prospective studies.

Cancer Registry data on the number and distribution of potential trial subjects could prove a valuable planning aid to future large trials. Investigators should be aware that raw incidence data are a poor general indicator of the number suitable for entry into even relatively 'open' protocols such as BSCG-1. Estimates can be improved by considering the effect of all the proposed entry criteria. The interpretation of trial results in the context of the population being treated would require similar care in matching suitable cases. Registry data are subject to many different confounding factors and should not be used as an alternative to trials when comparing treatment differences (Green & Byar, 1984). There is however a growing body of epidemiological evidence to suggest than patients treated according to trial protocols or by specialist centres do better that those managed elsewhere. These reports come from childhood cancer centres (Stiller, 1989) and in the rare adult tumours where major advances have been made, such as multiple myeloma (Karjalainen & Palva, 1989) and testicular cancer (Bagshawe *et al.*, 1985). There is still little evidence that outcome in the common adult solid tumours is improved by treatment at specialist centres (Stiller, 1992). Recent work looking at the management of breast and prostatic cancer in Finland confirms the difficulty of attributing observed variations in survival to differences in patterns of care (Karjalainen, 1990).

It is disappointing that in this study, 2 years of intensive follow-up by specialist trial clinics was not translated into improved survival over the concurrent non-trial cases. Other large series from the West Midlands Registry have shown that increased surgical experience can significantly reduce post operative mortality in resectable gastric and esophageal cancer, but long term outcome was not improved in those who survived operation (Allum *et al.*, 1989*b*; Matthews *et al.*, 1986). Until more effective treatments become available, early diagnosis and improved surgical management will continue to dominate the outcome in gastric cancer. There is a clear need to continue to test potential adjuvant treatments for this disease within the context of properly controlled and executed clinical trials.

We are indebted to the members of the British Stomach Cancer Group (listed in Fielding *et al.*, 1983), and the West Midlands Cancer Registry for the use of their data. We thank the Cancer Research Campaign, Medical Research Council and United Birmingham Hospitals Endowment fund for support. The work was carried out at the West Midlands Cancer Registry and the Cancer Research Campaign Trials Unit. The advice and assistance given by Ms Jean Powell, Mr M. Hallisey and Dr M. Cullen has been invaluable.

### References

ALLUM, W.H., HALLISEY, M.T. & KELLY, K.A. (1989*a*). Adjuvant chemotherapy in operable gastric cancer: 5 year follow-up of first British Stomach Cancer Group Trial. *Lancet*, **i**, 571–574.

ALLUM, W.H., POWELL, D.J., McCONKEY, C.C. & FIELDING, J.W.L. (1989*b*). Gastric cancer: a 25 year review. *Br. J. Surg.*, **76**, 535–540.

BAGSHAWE, K.D., BEGENT, R.H.J., NEWLANDS, E.S. & RUSTIN, G.J.S. (1985). What sort of oncology team should treat testicular teratoma? *Lancet*, **i**, 930.

BATES, T., RILEY, D.L., HOUGHTON, J., FALLOWFIELD, L. & BAUM, M. (1991). Breast cancer in elderly women: a Cancer Research Campaign trial comparing treatment with tamoxifen and optimal surgery with tamoxifen alone. The Elderly Breast Cancer Working Party. *Br. J. Surg.*, **78**, 591–594.

BAUM, M., ZILKHA, K. & HOUGHTON, J. (1989). Ethics of clinical research: lessons for the future. *Br. Med. J.*, **299**, 251–253.

BEGG, C.B. & ENGSTROM, P.F. (1987). Eligibility and extrapolation in cancer clinical trials. *J. Clin. Oncol.*, **5**, 962–968.

BEGG, C.B., ZELEN, M., CARBONE, P.P., McFADDEN, H.T., BRODOVSKY, H., ENGSTROM, P., HATFIELD, A., INGLE, J., SCHWARTZ, B. & STOLBACH, L. (1983). Cooperative groups and community hospitals: measurement of impact in the community hospitals. *Cancer*, **52**, 1760–1767.

BROOKES, V.S., WATERHOUSE, J.A.H. & POWELL, J. (1965). Carcinoma of the stomach: 10 year survey of results and of factors affecting prognosis. *Br. Med. J.*, **1**, 1577–1583.

BYAR, D.P. (1980). Why databases should not replace randomised clinical trials. *Biometrics*, **36**, 337–342.

BYAR, D.P. (1988). The use of data bases and historical controls in treatment comparisons. *Recent Results Cancer Res.*, **111**, 95–98.

CALIFF, R.M., PRYOR, D.B. & GREENFIELD, J.C. (1986). Beyond randomised clinical trials: applying clinical experience in the treatment of patients with coronary heart disease. *Circulation*, **74**, 1191–1194.

CASS PRINCIPLE INVESTIGATORS AND THEIR ASSOCIATES (1984). Coronary Artery Surgery Study (CASS): a randomised trial of coronary artery bypass surgery. Comparability of entry characteristics and survival in randomised and nonrandomised patients meeting randomisation criteria. *J.A.C.C.*, **3**, 114–128.

CHALMERS, T. (1990). Ethical implications of rejecting patients for clinical trials (editorial). *JAMA*, **263**,865.

CHARLSON, M.E. & HOROWITZ, R.I. (1984). Applying results of randomised trials to clinical practice: impact of losses before randomisation. *Br. Med. J.*, **289**, 1281–1284.

CURTIS, R.E., KENNEDY, B.J., MYERS, M.H. & HANKEY, B.F. (1985). Evaluation of AJC stomach staging using the SEER population. *Semin. Oncol.*, **12**, 21–31.

DAVIS, K. (1988). The comprehensive cohort study: the use of registry data to confirm and extend a randomised trial. *Recent Results Cancer Res.*, **111**, 138–148.

DIXON, W.J., BROWN, M.B., ENGLEMAN, L., HILL, M.A. & JENNRICH, R.I. (1988). BMDP Statistical Software Manual, University of California Press: Berkley.

ELWOOD, P.C. (1982). Randomised controlled trials: sampling. *Br. J. Clin. Pharmacol.*, **13**, 631–636.

EXTRAMURAL COMMITTEE TO ASSESS MEASURES OF PROGRESS AGAINST CANCER (1990). Measurement of progress against cancer. *J. Natl Cancer Inst.*, **82**, 825–835.

FENTIMAN, I.S., JULIEN, J.P. VAN, D.J., VAN, G.B., CHETTY, U. & COIBION, M. (1991). Reasons for non-entry of patients with DCIS of the breast into a randomised trial (EORTC 10853). *Eur. J. Cancer*, **27**, 450–452.

FIELDING, J.W.L. (1989*a*). Cancer of the stomach: demographic aspects. In Fielding, J.W.L., Powell, J., Allum, W.H., Waterhouse, J.A.H. & McConkey, C.C. (eds), *Cancer of the Stomach* pp. 12–41. Macmillan Press: Southampton.

FIELDING, J.W.L., FAGG, S.L., JONES, B.G., ELLIS, D., HOCKEY, M.S., MINAWA, A., BROOKES, V.S., CRAVEN, J.L., MASON, M.C., TIMOTHY, A., WATERHOUSE, J.A.H. & WRIGLEY, P.F.M. (1983). An interim report of a prospective, randomised, controlled study of adjuvant chemotherapy in operable gastric cancer: British Stomach Cancer Group. *World J. Surg.*, **7**, 390–399.

FIELDING, J.W.L., POWELL, J., ALLUM, W.H., WATERHOUSE, J.A.H. & MCCONKEY, C.C. (1989). *Cancer of the Stomach*. Macmillan Press: Southampton.

FRIEDMAN, M.A. & CAIN, D.F. (1990). National Cancer Institute sponsored cooperative clinical trials. *Cancer*, **65**, 373–379.

GEHAN, E.A. (1980). Use of prognostic factors in analysis of historical control studies. *Cancer Treat. Rep.*, **64**, 2376–2382.

GEHAN, E.A. & FREIREICH, E.J. (1974). Non-randomised controls in cancer clinical trials. *N. Engl. J. Med.*, **290**, 198–203.

GREEN, S.B. & BYAR, D.P. (1984). Using observational data from registries to compare treatments: the fallacy of omnimetrics. *Stat. Med.*, **3**, 361–370.

HUNTER, C.P., FRELICK, R.W., FELDMAN, A.R., BAVIER, A.R., DUNLAP, W.H., FORD, L., HENSON, D., MACFARLANE, D., SMART, C.R., YANCIK, R. & YATES, J.W. (1987). Selection factors in clinical trials: results from the Community Clinical Oncology Program physicians patient log. *Cancer Treat. Rep.*, **71**, 559–565.

KAPLAN, E.L. & MEIER, P. (1958). Nonparametric estimation from incomplete observation. *J. Am. Stat. Assoc.*, **53**, 457–481.

KARJALAINEN, S. (1990). Geographical variation in cancer patient survival in Finland: chance, confounding, or effect of treatment? *J. Epidemiol. Community Health*, **44**, 210–214.

KARJALAINEN, S. & PALVA, I. (1989). Do treatment protocols improve end results? A study of survival of patients with multiple myeloma in Finland. *Br. Med. J.*, **299**, 1069–1072.

LEE, J.Y. & BREAUX, S.R. (1983). Accrual of radiotherapy patients to clinical trials. *Cancer*, **52**, 1014–1016.

MANTEL, N. (1983). Cautions of the use of medical databases. *Stat. Med.*, **2**, 355–362.

MARTIN, J.F., HENDERSON, W.G. & ZACHARSKI, L.R. (1981). Accrual of patients into a multihospital cancer clinical trial and its implications on planning future studies. *Controlled Clin. Trials*, **2**, 74.

MATTHEWS, H.R., POWELL, D.J. & MCCONKEY, C.C. (1986). Effect of surgical experience on the results of resection for oesophageal cancer. *Br. J. Surg.*, **73**, 621–623.

MCCUSKER, J., WAX, A. & BENNETT, J.M. (1982). Cancer patient accessions into clinical trials: a pilot investigation into some patient and physician determinants of entry. *Am. J. Clin. Oncol.*, **5**, 277–236.

MCDONALD, C.J. & HUI, S.L. (1991). The analysis of humongous databases: problems and promises. *Stat. Med.*, **10**(4), 511–518.

PETO, R., PIKE, M.C., ARMITAGE, P., BRESLOW, N.E., COX, D.R., HOWARD, S.V., MANTEL, N., MCPHERSON, K., PETO, J. & SMITH, P.G. (1977). Design and analysis of randomised clinical trials requiring prolonged observation of each patient II. Analysis and Examples. *Br. J. Cancer*, **35**, 1–39.

POCOCK, S.J. (1976). The combination of randomised and historical controls in clinical trials. *J. Chron. Dis.*, **29**, 175–188.

STILLER, C. (1992). Survival of patients in clinical trials and at specialist centres. In Williams, C.J. (eds) *Introducing New Treatments for Cancer: Practical, Ethical and Legal Problems* pp. 119–136. John Wiley & Sons Ltd: Chichester.

STILLER, C.A. (1989). Survival of patients with cancer: those included in trials do better. *Br. Med. J.*, **299**, 1058–1059.

TATE, H.C., RAWLINSON, J.B. & FREEDMAN, L.S. (1979). Randomised comparative studies in the treatment of cancer in the UK: room for improvement? *Lancet*, **2**, 623–624.

TORONTO LEUKEMIA STUDY GROUP (1986). Results of chemotherapy for unselected patients with acute myeloblastic leukemia: effect of exclusions on interpretation of results. *Lancet*, **i**, 786–788.

WATERHOUSE, J.A.H., MUIR, C., CARREA, P. & POWELL, J. (1976). *Cancer Incidence in Five Continents*. IARC Scientific Publications: Lyon, France.

YUSEF, S., HELD, P., TEO, K.K. & TORETSKY, E.R. (1990). Selection of patients for randomised trials: implications of wide or narrow eligibility criteria. *Stat. Med.*, **9**, 73–86.